# Unraveling the role of rat and flea population dynamics on the seasonality of plague epidemics in Madagascar

**DOI:** 10.1073/pnas.2502161122

**Published:** 2025-06-12

**Authors:** Fanohinjanaharinirina Rasoamalala, Beza Ramasindrazana, Mamionah J. Parany, Soloandry Rahajandraibe, Lovasoa Randriantseheno, Soanandrasana Rahelinirina, Olivier Gorgé, Eric Valade, Mireille Harimalala, Minoarisoa Rajerison, Simon Cauchemez, Antoine Brault

**Affiliations:** ^a^Plague Unit, Institut Pasteur de Madagascar, Antananarivo 101, Madagascar; ^b^Doctoral School Life and Environmental Sciences, University of Antananarivo, Antananarivo 101, Madagascar; ^c^Department for the Control of Transmissible Diseases, Central Laboratory for Plague, Ministry of Public Health, Antananarivo 101, Madagascar; ^d^Institut de Recherche Biomédicale des Armées, Brétigny-sur-Orge 91220, France; ^e^Ecole du Val-de-Grâce, Paris 75005, France; ^f^Medical Entomology Unit, Institut Pasteur de Madagascar, Antananarivo 101, Madagascar; ^g^Mathematical Modelling of Infectious Diseases Unit, Global Health Department, Institut Pasteur, Université Paris Cité, INSERM U1332, CNRS UMR2000, Paris 75015, France

**Keywords:** plague, seasonality, public health, modeling, Madagascar

## Abstract

Plague continues to pose a public health problem in multiple regions of the world, including Madagascar, where it is characterized by a pronounced seasonal pattern. The drivers of plague seasonality remain poorly understood. Using a deterministic compartmental model, calibrated to rat and flea capture data, serological data collected in active rural foci, and human plague surveillance data, we analyzed the effects of seasonal rat and flea population dynamics on plague transmission. The models that incorporated seasonal fluctuations in rat and flea populations provided better predictive performances than those that did not. We found that a simpler mass-action model also performed well. Driven by these seasonal changes, the effective reproduction number (R_e_) between rats peaks at 1.45 [95% credible interval (CI): 1.41, 1.48] in October and falls to 0.6 (95% CI: 0.57, 0.63) in March. We estimated that 0.5% (95% CI: 0.2%, 0.9%) of rats are infected annually, indicating that plague is not the main driver of rat population changes. Using our model, we evaluated intervention strategies and found that targeting both rats and their fleas at the start of the epidemic season (July–September) was the most effective approach for reducing human plague cases. Such an approach contrasts with the reactive strategy currently employed in Madagascar. Our findings highlight the role of flea and rat populations in plague seasonality and identify strategies that could be deployed in Madagascar to better control plague epidemics.

## Significance

Plague epidemics in Madagascar show a seasonal pattern that continues to pose public health challenges. By combining field data on rat and flea populations with mathematical modeling, we unveil how seasonal changes in these populations drive human plague epidemics. Using this model, we investigate several plague control strategies. We find that targeting both rat populations and their associated fleas at the onset of the epidemic season is the most effective strategy for reducing human plague cases, though controlling either rats or fleas alone also yields positive results. Compared to the reactive strategies currently employed in Madagascar, our results suggest that a preventive approach may be more effective. Our modeling can help decision-makers design a roadmap to mitigate future plague epidemics.

**Fig. 1.**
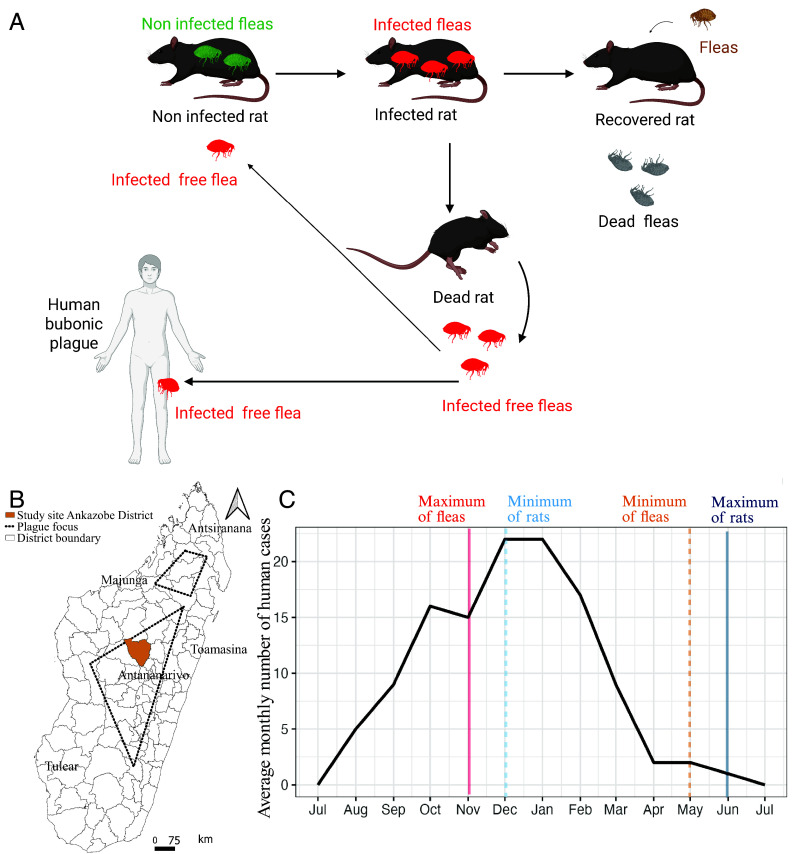
Plague in Madagascar. (*A*) Plague transmission cycle that shows the interactions between rats, vector fleas, and humans. The cycle includes susceptible rats infested with uninfected fleas; rats infected by infected fleas; rats that die from plague, releasing infected fleas into the environment; and recovered rats, from which infected fleas die off, though infestation by fleas (infected or uninfected) may persist. (*B*) Map highlighting the regions of Madagascar where plague is endemic (dotted outlines). The study site in the Ankazobe District is marked in yellow. (*C*) Average monthly number of human plague cases in Madagascar between 2018 and 2023.

Despite significant control efforts, plague continues to pose a persistent public health challenge in several regions of the world. Between 2013 and 2018, approximately 2,800 human cases were reported globally (1), with a case fatality ratio of 17.5%. Plague is a vector-borne zoonotic disease caused by the gram-negative bacterium *Yersinia pestis* (2). This pathogen has various natural hosts among vertebrates in particular small mammals (3), and it is mainly transmitted to humans by the bite of an infected flea, resulting in bubonic plague (Fig. 1*A*). If left untreated, this can progress to septicemic or pulmonary plague, with high mortality rates. In addition, the pulmonary form presents a risk of human-to-human transmission (2).

In Madagascar, which reported 80% of the world’s plague cases between 2013 and 2018 (1), the majority of cases occur in rural areas of the Central Highlands, with a few isolated cases occasionally reported in the north and on the west coast of the island, forming plague foci (4–6) (Fig. 1*B*). In the Central Highlands, the circulation of plague in these foci follows a marked seasonal pattern, with a peak of incidence in humans during the hot and humid season from September to April (4, 5) (Fig. 1*C*). Rat and flea populations also exhibit seasonal fluctuations, with rat abundance peaking in June and July (7, 8) when human cases are rare (5) and flea abundance peaking between October and November (9), just prior to the average peak in human cases from December to January (4, 5) (Fig. 1*C*).

A larger flea population may amplify plague transmission among rats and to humans, which could explain why the rise in flea numbers coincides with the peak in human cases (9). Conversely, although a decline in the rat population may reduce plague transmission among rats, the resulting scarcity of rat hosts might drive fleas to seek human hosts, thereby increasing the risk of plague for humans (9, 10). These processes could account for the observed rise in human cases during December and January, yet the precise mechanisms shaping the timing and amplitude of human outbreaks remain poorly characterized.

A better understanding of these mechanisms is crucial to optimize plague control strategies. Indeed, during epidemics, the response led by Madagascar’s Ministry of Public Health includes the use of insecticides recommended by the WHO, as well as mass capture of live rats, in order to prevent the dispersal of fleas. However, local populations also use rodenticides and snap traps, which may inadvertently worsen outbreaks by driving fleas from dead rats to seek new hosts.

The objective of this study was to characterize the relationship between rat and flea seasonality and periods of high plague transmission in humans, in plague foci in the Central Highlands of Madagascar. To identify the mechanisms that provided the best explanation for plague seasonality, compartmental models describing different potential mechanisms were calibrated to detailed field data, including rat and flea capture surveys and associated rat serology collected between December 2018 and June 2020, in the Central Highlands (9) as well as human plague surveillance for Madagascar, 2018 to 2023. The model was then used to assess which control strategy was expected to minimize the number of human plague cases.

## Results

### Plague Data.

**Fig. 2.**
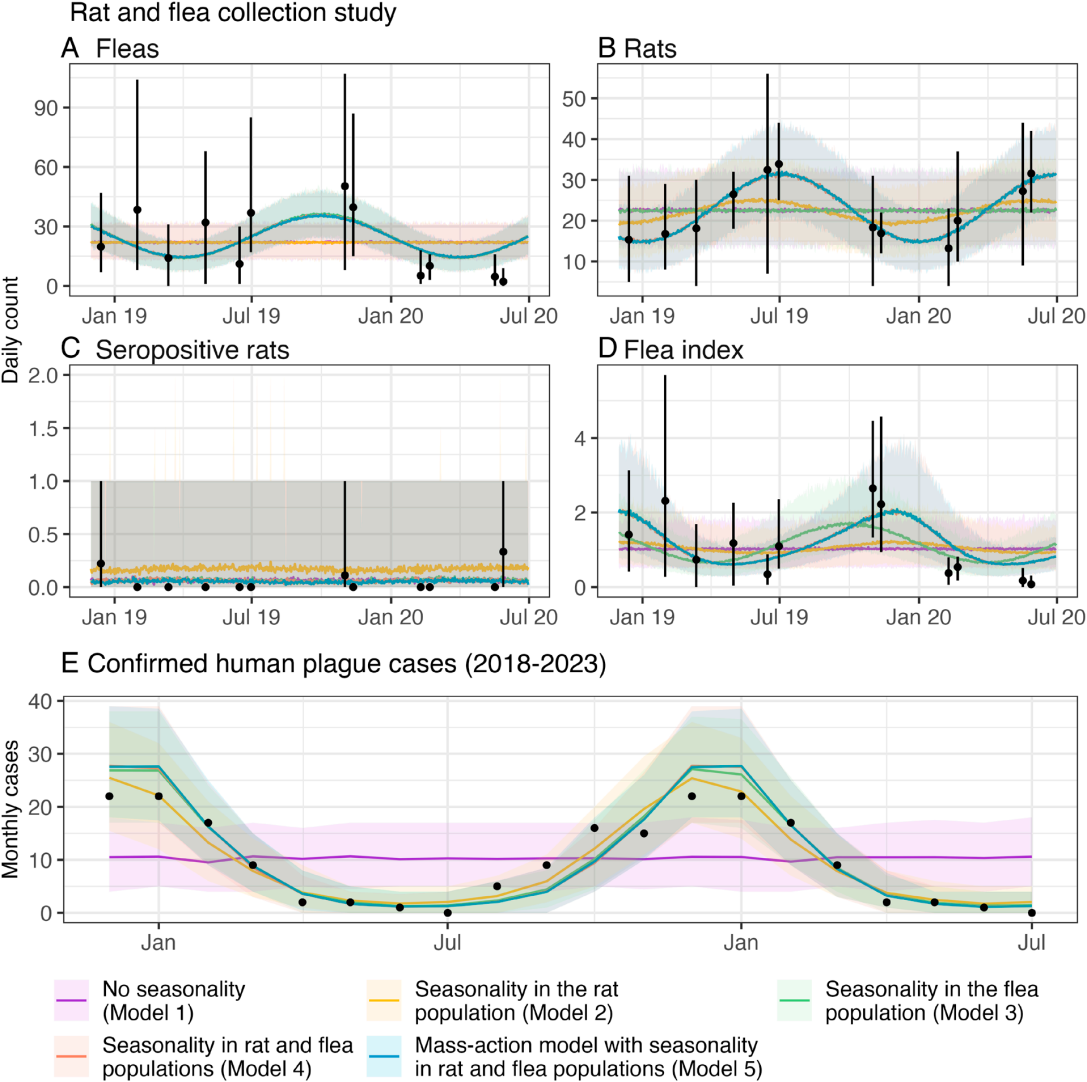
Model calibration. Comparison of data and model predictions for (*A*) the number of collected fleas, (*B*) the number of collected rats, (*C*) the number of collected plague seropositive rats, (*D*) the flea index (mean number of fleas per rat), (*E*) average monthly number of confirmed human plague cases between 2018 and 2023. For Figures *A*–*D*, black dots represent the median values of data aggregated temporally proximate capture days, with vertical lines indicating the range between the minimum and the maximum of observed values across those days. For Figure *E*, black dots represent the monthly average number of confirmed human plague cases in the data. The models include no seasonality (Model 1 – purple), seasonality in the rat population (Model 2 – yellow), seasonality in the flea population (Model 3 – green), seasonality in both rat and flea populations (Model 4 – red), and mass-action model with seasonality in both rat and flea populations (Model 5 – blue).

Between December 2018 and June 2020, sessions to capture small mammals and their fleas were carried out six times in each of the six communes in the district of Ankazobe, Madagascar. A total of 2,432 rats (*Rattus rattus*) were captured across the different sessions conducted in the villages of the Ankazobe district (Fig. 1*B*). Of these, 410 individuals were trapped inside houses, while 2,022 were captured outside. A seasonal peak in flea abundance, defined as the total number of fleas collected from rats during sampling, was observed in November (Fig. 2*A*), while rats showed a distinct seasonal pattern, with their population peaking in June-July (Fig. 2*B*). Six rats were seropositive (0.2%; 2,432 individuals tested), revealing previous exposure to *Y. pestis* infection (Fig. 2*C*). The flea index, which represents the mean number of fleas per rat, reaches a peak in November (Fig. 2*D*). Between 2018 and 2023, 695 confirmed human plague cases were reported throughout Madagascar, with an annual number of cases ranging from 53 to 150 and a peak occurring between October and January (*SI Appendix*, Fig. S1). Over this period, the average monthly numbers of human plague cases show a pronounced peak between December and January (Fig. 2*E*).

### Models Reproducing Plague Seasonal Patterns.

To evaluate the role of rat and flea population seasonality in plague epidemics, we started by fitting four models adapted from Keeling et al. (10, 11). In those models, the rat population is divided into susceptible, infected, and immune categories, and the flea population into hosted fleas on rats and free infected fleas. They incorporate a mechanism, where the density of the rat population $N_{r}$ influences the host-finding success of free infected fleas. The probability that a free infected flea finding a rat host is formulated as $1-exp(-bN_{r})$, where b is the flea search index. This probability decreases when the rat population $N_{r}$ decreases. Conversely, the probability of a flea not finding a rat (potentially leading it to target humans) is $exp(-bN_{r})$, which increases as $N_{r}$ decreases. Model parameters such as the flea search index were calibrated using a Bayesian approach to data on rat and flea counts, seropositive rats, and the monthly average of human plague cases from 2018 to 2023. Model 1, which did not take into account the seasonality of flea and rat populations, failed to capture the seasonality of human plague epidemics (Fig. 2*E*). It had the highest leave-one-out cross-validation (LOO-CV) score of 4,951, meaning the lowest predictive performance (*SI Appendix*, Table S1). Model 2, which incorporated rat population seasonality, performed better (LOO-CV: 4,465). While it explained variations in human cases (Fig. 2*E*), it failed to capture variation in the flea population (Fig. 2*A*) and underestimated the amplitude of the flea index (Fig. 2*D*). Model 3, which included seasonal fluctuations in the flea population (but not in the rat population), better explained the flea index amplitude (Fig. 2*D*) and captured seasonal variations in human epidemics (LOO-CV: 4,209) (Fig. 2*E*). The model’s predictive performance improved substantially (LOO-CV: 4,028) when both rat and flea population dynamics were incorporated (Model 4, Fig. 2).

### Mass-Action Model.

Our estimates indicate that the flea search index is small [6.7e-7 (95% CI: 1.7e-8, 2.4e-6)], leading to a low product $bN_{r}$ [5.8e-3 (95% CI: 2.6e-4, 5.8e-2)]. Consequently, variations in the rat population do not substantially affect the proportion of free fleas infecting humans. Accordingly, we explored a simpler mass-action model (Model 5) in which infection rates for susceptible rats and humans are directly proportional to the number of free infected fleas and no longer depend on the flea search index. This model demonstrated a slight improvement, with a LOO-CV score of 4,025 compared to 4,028 for Model 4 (Fig. 2 and *SI Appendix*, Table S1).

### Plague Epidemic in the Rat Population.

**Fig. 3.**
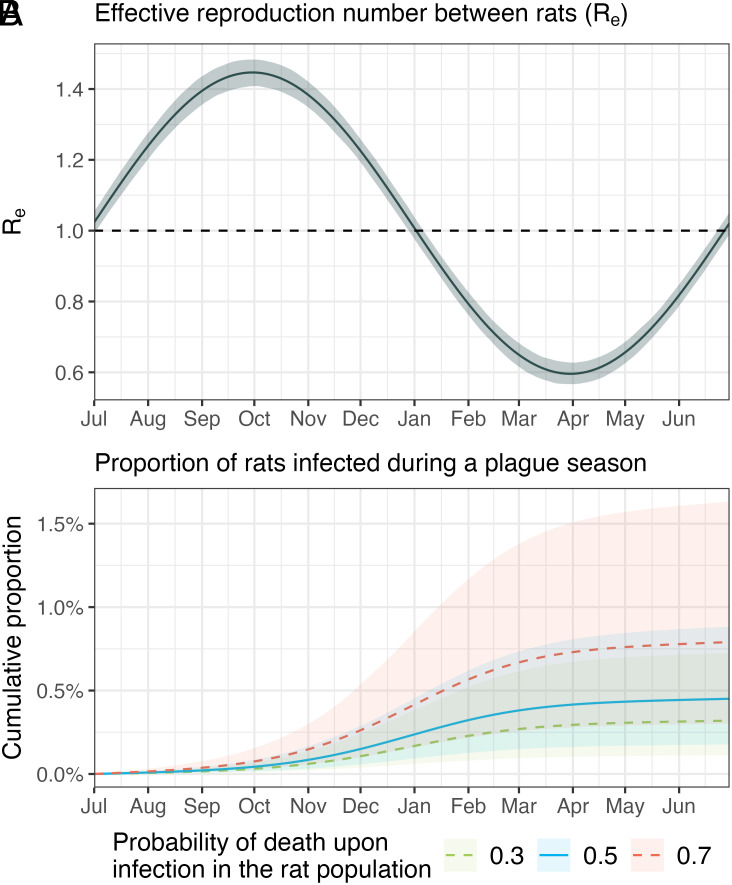
Plague epidemic in the rat population. (*A*) Effective reproduction number (Re) among rats, with the blue solid line showing the estimated R_e_ over time, and the black dashed horizontal line representing the epidemic threshold (R_e_ = 1). (*B*) Cumulative proportion of infected rats throughout a plague season from July 1 to June 30, assuming a probability of death upon infection of 0.3 (green dashed line), 0.5 (blue solid line, representing the baseline scenario), and 0.7 (red dashed line).

Using the best-performing model, Model 5 (which includes rat and flea seasonalities), we reconstructed plague dynamics in rats. The effective reproduction number, $R_{e}$, among rats rises above 1 at the beginning of July, initiating the seasonal plague epidemic within rat populations, peaks at 1.45 (95% CI: 1.41, 1.48) in October, falls below 1 by mid-January, and reaches a minimum of 0.6 (95% CI: 0.57, 0.63) by late March (Fig. 3*A*). Over an entire plague season from July 1 to June 30, we estimated a low cumulative infection rate within the rat population, between 0.5% (95% CI: 0.2%, 0.9%), under the baseline scenario with an assumed probability of rat death upon plague infection of 0.5 (12, 13) (Fig. 3*B*). The cumulative infection rate varied between 0.3% (95% CI: 0.1%, 0.7%) and 0.8% (95% CI: 0.3%, 1.6%), when changing the probability of rat death upon infection to 0.3 and 0.7, respectively (Fig. 3*B*). This variation arises because a higher infection fatality rate requires a larger number of infected rats to account for the observed seropositivity among surviving rats in the data.

### Impact of Interventions to Reduce Human Cases.

We evaluated three intervention strategies, applied to plague reservoirs and vectors, aimed at mitigating the spread of bubonic plague in humans: 1) controlling only the rat population, using methods like rodenticides or snap traps, which leave fleas free to seek new hosts, modeled as a reduction in the rat population; 2) targeting fleas directly, using insecticides to reduce their populations, modeled as a reduction in both the flea population hosted on rats and the free infected fleas; and 3) simultaneously targeting rat populations and the fleas hosted on those rats, achieved through methods like live-capturing rats and collecting their fleas to prevent escape, or using a combination of rodenticides and insecticides, modeled as a proportional reduction in both the rat population and hosted fleas. We evaluated several intervention start dates, set at the beginning of each month from July 1 to March 1, and assessed their impact based on the proportion of human cases averted over the plague season spanning July 1 to June 30.

**Fig. 4.**
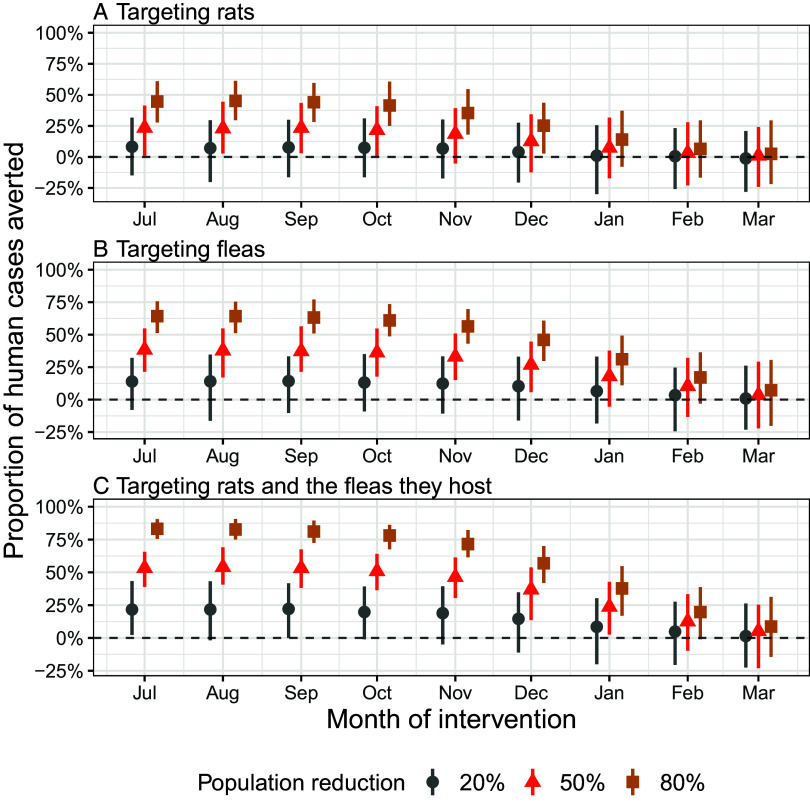
Impact of the control of rat and flea populations on the number of human plague cases. The figures show the proportion of human cases averted over one season (July 1 to June 30) as a function of the month of intervention when (*A*) only rats are targeted, (*B*) only fleas are targeted, and (*C*) both rats and the fleas on those rats are simultaneously targeted. Reduction levels are represented by gray dots (20% population reduction), red triangles (50% population reduction), and yellow rectangles (80% population reduction). Vertical bars indicate the 95% credible intervals.

The results showed that, regardless of the type of intervention, reducing the target populations led to a greater decrease in the number of human cases (Fig. 4). Interventions targeting fleas were more effective than those targeting rats alone (Fig. 4 *A* and *B*). The combined approach, targeting both rats and their hosted fleas, resulted in the greatest reduction in human cases (Fig. 4*C*). The impact remained unchanged when the interventions started in September instead of July. However, impact declined if implementation was moved later in the season, for example 50% reductions in rat and flea populations reduce plague human cases by 5% (95% CI: −23%, 25%) when implemented in March vs 53% (95% CI: 38%, 67%) for an implementation in September (Fig. 4*C* and *SI Appendix*, Table S2).

## Discussion

Our findings emphasize the pivotal influence of seasonal rat and flea population dynamics on human plague epidemics and detail how such understanding of the drivers of plague seasonality may help design a roadmap to mitigate future plague epidemics.

Previous plague modeling studies have primarily focused on mechanisms of plague persistence, disappearance, and sporadic human epidemics (10, 11, 14, 15). The influence of rat and flea population dynamics on seasonal human plague incidence, however, remains understudied. By integrating detailed field data on fleas, rats, and humans, we show that the timing of human cases can be explained by the population dynamics of rats and fleas.

Keeling et al. (10, 11) also suggested that declining rat populations could elevate human risk by prompting infected fleas to seek alternate hosts. However, our simpler mass-action model (Model 5), which omits this mechanism, performs slightly better, indicating that the flea search dynamics may not be essential to replicate observed outbreak patterns. Additional studies could provide more information about how fleas adjust their behavior in response to a decline in rat host populations.

Thanks to serological data on rats, we showed that the proportion of rat population infected each plague season is under 1.6%. In contrast, the rat population decreases by 53% (95% CI: 47 to 58%) between its annual maximum and minimum (Fig. 2*B*). This demonstrates that the rat population is not primarily driven by plague epidemics. Rather, its variations can be explained by ecological factors, notably access to food resources conducive to their reproduction, linked to environmental variations such as the nature of habitats (inside and outside houses, rice fields, sisal areas), agricultural activities, as well as methods of storing foodstuffs in dwellings at certain times of the year. A population peak is generally observed between July and August, following strong reproduction in March, which coincides with the harvest season and maximum availability of food resources (7, 8, 16). In addition, it has been shown that flea dynamics are strongly influenced by climatic conditions (temperature and humidity) at all stages of their development, with a peak in abundance in November (5, 9, 17). We provided insights into how periods of high flea abundance contribute to the intensification of plague transmission to humans.

Current plague control strategies, implemented by the Madagascar Ministry of Health, are based on multilevel interventions. These include early diagnosis for rapid treatment of confirmed cases, investigation of suspected and at-risk individuals, and surveillance actions to reduce reservoirs (rats) and vectors (fleas) in order to limit the transmission and spread of the infection (5). These interventions include flea control campaigns using appropriate insecticides, combined with the capture of small mammals to reduce their populations and biological analysis of the animals and their fleas. However, these strategies remain essentially reactive, generally being implemented after confirmation of human cases. Our results suggest that a proactive strategy aimed at reducing rat and flea populations at the onset of the epidemic season between July and September could be effective in reducing the incidence of human plague. Such an approach, oriented toward prevention and planned on a seasonal basis, might prove more effective than current interventions, which are often triggered only after the appearance of human cases. By integrating preventive and seasonal planning, the fight against plague could be significantly strengthened, with a lasting reduction in its impact on populations.

Our study has some limitations. First, due to the lack of a sufficiently large spatially resolved dataset, we calibrated a deterministic model to the average human plague cases across Madagascar, whereas plague epidemics usually occur locally. Access to the same type of collection survey on a large area over a prolonged period could enable a direct correlation between human cases, rats, and flea dynamics, allowing for a more precise understanding of the mechanisms driving plague transmission at the local level. Second, the persistence of plague in our model results from a balance of parameters chosen during the fitting process, which maintains a low but consistent level of infection between seasons. Although this approach successfully reproduces the observed seasonal dynamics, it does not fully capture the deeper mechanisms underlying plague maintenance. In particular, incorporating spatial structure and host heterogeneity such as variations in rat susceptibility or resistance coupled with detailed ecological and epidemiological data could offer a better understanding of plague persistence (10, 15). Third, our model does not include the detailed life cycles of fleas and rats due to a lack of data. Integrating future data on these life cycles in a model could help refine the model and identify more precise optimal timings for interventions. Finally, the probability of death upon infection for *R. rattus* appears to be heterogeneous in Madagascar, with lower mortality in endemic zones compared to nonendemic zones potentially explained by genetic selection (12, 13, 18). To address this uncertainty, we conducted a sensitivity analysis to capture the range of plausible mortality rates within rat populations.

This study underscores the importance of collecting field data on plague vectors, which, when combined with modeling approaches, provide valuable insights into human plague epidemics and inform the optimization of mitigation strategies. Future research prioritizing collecting more comprehensive data of this nature will help to refine models and improve public health strategies in endemic areas.

## Material and Methods

### Data on Rat, Fleas, and *Y. pestis* Detection.

From December 6, 2018, to June 3, 2020, small mammal captures and flea collections were conducted in six villages in the Ankazobe district. In each village, 120 live-wire traps were deployed inside and outside houses for three consecutive nights during each capture session. These capture sessions were repeated six times in each village, giving a total of six series of captures per village (sessions 1 to 6) to capture seasonal variations in populations.

The small mammals captured and the fleas collected were specifically identified and then subjected to a series of analyses to detect the presence of plague bacterium. These analyses included bacteriological tests using culture and inoculation on mice (5), followed by molecular analysis through qPCR tests targeting the “*caf*” and “*pla*” genes (19), as well as serological analyses using a direct ELISA test to detect the anti-F1 antibody specific to *Y. pestis* (20). A total of 2,762 small mammals were captured, with 2,432 (88%) of these being *R. rattus*. We focused our study on *R. rattus*, the most abundant rodent population collected (21), also known to be the primary vector of plague transmission to humans (6, 8), excluding other small mammal species. Additionally, 2,464 fleas were collected, 99.8% of which were identified as two plague vector species: *Synopsyllus fonquerniei* (N = 1,334; 54.1%) and *Xenopsylla cheopis* (N = 1,125; 45.7%). The remaining 0.2% consisted of other species: *Pulex irritans* and *Ctenocephalides felis*.

### Data on Human Cases and Diagnosis.

A surveillance system has been set up within the Central Plague Laboratory, based at the Plague Unit of the Institut Pasteur de Madagascar, to deal with all cases of plague reported in Madagascar (*SI Appendix*, Fig. S1). Each suspected case of human plague is notified as soon as clinical suspicion is established. All samples taken are analyzed using various tests, including the rapid diagnostic test, bacteriology and qPCR (5, 19, 20). The results are interpreted according to the World Health Organization’s case definitions, with a confirmed case of plague defined as having a positive result from either a PCR test, bacteriological culture, or both (22).

### Mathematical Model.

We adapted the deterministic compartmental model developed by Keeling et al. (10) to describe the plague transmission dynamics in rat, flea and human populations incorporating seasonal variations of these populations (*SI Appendix*, Fig. S2). At time *t*, the rat population is divided into susceptible ($S_{r}(t)$), infected ($I_{r}(t)$), and immunized ($R_{r}(t)$) compartments, while the flea population is categorized into fleas hosted on rats (*H*(*t*), including both infected and noninfected) and free infected fleas (*F*(*t*)), which are not on a host and actively searching for one. We denote $N_{r}(t)$= $S_{r}(t)$ +$I_{r}(t)$ + $R_{r}(t)$ the total number of rats. The probability that a free flea finds a rat host is parameterized as $1-exp(-bN_{r}\left(t\right)$), where the flea search index b is an estimated parameter. Then, denoting β the estimated transmission rate between rats and fleas, the number of newly infected rats during a small time interval dt, is given by $\beta \;\left(S_{r}\left(t\right)/N_{r}\left(t\right)\right)\;(1-exp\;\left(-bN_{r}\left(t\right)\right))\;F\left(t\right)\;dt$. Infected rats remain infectious for an average period of 1/γ=7 d (23). They die from plague infection with probability η=0.5 (12) or become immune for an average period of 1/ω=1 y (24) with probability 1-η=0.5. We conducted a sensitivity analysis using η values of 0.3 and 0.7.

When a rat dies from plague, $H(t)/N_{r}(t)$ infected fleas hosted on it are released. These free fleas live for an average period of 1/ϕ=10 d (10, 24) during which they actively search for a new host. Rat and flea populations are modeled by fixed growing rate computed as the difference between reproduction rates (average number of offspring produced by an individual per year) and mortality rate $r_{r}=35-1=34$ y^−1^ (23, 25) and $r_{f}=140-12/6=138$ year^−1^ (26) respectively. The force of infection in the human population is assumed to be proportional to the number of free infected fleas that do not find a rat host: $\lambda_{h}=a\;F\left(t\right)\;exp\left(-bN_{r}\left(t\right)\right),$ where a is estimated.

The evolution of the population in each compartment, at time t, is then given by the following differential equations:

[1] $$\begin{array}{c} dS_{r}\;(t)/dt=r_{r}\;S_{r}(t)\;(1-N_{r}\;(t)/K_{r}(t))\\ \;-\beta \;(S_{r}\;(t)/N_{r}(t))\;(1-exp\;(-b\;N_{r}(t)))\;F(t)+\omega R_{r}(t), \end{array}$$

[2] $$\begin{array}{c} dI_{r}(t)/dt=\beta \;(S_{r}(t)/N_{r}(t))\;(1-exp\;(-b\cdot N_{r}(t)))\;F(t)-\gamma \;I_{r}(t), \end{array}$$

[3] $$dR_{r}\;(t)/dt=\gamma \;(1-\eta )\;I_{r}\;(t)-\omega \;R_{r}\;(t),$$

[4] $$\begin{array}{c} dH\;(t)/dt=r_{f}\;H(t)\;(1-H\;(t)/K_{f}(t))-\eta \;\gamma \;I_{r}\;(t)\;H(t)/N_{r}\;(t), \end{array}$$

[5] $$\begin{array}{c} dF\;(t)/dt=\eta \;\gamma \;I_{r}\;(t)\;H(t)/N_{r}\;(t)-\phi \;F\;(t), \end{array}$$

[6] $$\lambda_{h}=a\;F\;(t)\;exp(-b\;N_{r}(t)),$$

where $K_{r}\left(t\right)=\left(A_{r}\left(cos\left(2\pi \left(t-t_{r}\right)/365.25\right)-1\right)/2\;+1\right)B_{r}$ and $\begin{array}{c} K_{f}\left(t\right)= \end{array}$ $\begin{array}{c} {(A}_{f}\left(cos\left(2\pi \left(t-t_{f}\right)/365.25\right)-1\right)/2+1)\;B_{f} \end{array}$ are the carrying capacities of the rat and flea populations, respectively. The parameters $B_{r}$, $B_{f}$ are the minimum capacities, $A_{r}$, $A_{f}$ are the relative amplitude of the seasonal fluctuations, and $t_{r}$ and $t_{f}$ indicate the timing of the peak capacities. Consequently, the maximum capacities for the rat and flea populations are $\left(A_{r}+1\right)B_{r}$ and $\left(A_{f}+1\right)B_{f}$, respectively. Although fleas feed on rat blood, we modeled flea population capacity independently of rat population capacity, as scientific literature suggests that climatic conditions have a greater influence on flea populations than host availability (9, 17, 27).

In the mass-action model, Eqs. **2** and **6** simplify to $dI_{r}\left(t\right)/dt=$ $\beta \;S_{r}(t)\;F(t)-\gamma \;I_{r}\left(t\right)$ and $\lambda_{h}=a\;F(t)$.

Models were coded using the *odin 1.5.10* R package.

We ran models starting from January 1, 1930, to allow them to reach a stable state before comparing them to the data from December 2018.

### Assumptions on Rat and Flea Populations Dynamics.

To understand the mechanisms that lead to seasonal plague epidemics in humans, we tested the following five models:

1. no seasonality in flea and rat population ($A_{f}=0,A_{r}=0$)
2. seasonality only in the rat population and no seasonality in the flea population ($A_{f}=0$)
3. seasonality only in the flea population and no seasonality in the rat population ($A_{r}=0$)
4. seasonality in rat and flea populations
5. mass-action model with seasonality in rat and flea populations

### Effective Reproduction Number.

We define the effective reproduction number $R_{e}\left(t\right)$ at time t in rats as the number of new rats that one infectious rat infects. In Models 1 to 4, one infectious rat releases $\eta \;H\left(t\right)/N_{r}\left(t\right)$ free infected fleas, and each flea infects $\beta S_{r}\;(t)\;(1-exp\;(-bN_{r}\left(t\right)))/\left(\phi N_{r}\left(t\right)\right)$ rats, it turns out that $\begin{array}{c} R_{e}\left(t\right)=\beta \;\eta \;S_{r}\left(t\right)\;(1-exp\;\left(-bN_{r}\left(t\right)\right))\;H\left(t\right)/\left(\phi {N_{r}}^{2}\left(t\right)\right) \end{array}$. In Model 5, using similar reasoning, the effective reproduction number simplifies to $R_{e}\left(t\right)=\beta \;\eta \;S_{r}\left(t\right)H\left(t\right)/\left(\phi N_{r}\left(t\right)\right)$.

### Model Calibration.

The model was fitted to the count of captured fleas and rats, as well as the proportion of seropositive rats. The likelihood is

$$\begin{array}{c} \prod_{t=1}^{n_{\mathrm{cap}}}g\left(r_{\mathrm{cap}}\left(t\right)|\rho \;N_{r}(t)\right)\;g\left(f_{\mathrm{cap}}\left(t\right)|\rho \;H\left(t\right)\right)\;g\left(r_{\mathrm{sero}}\left(t\right)|\rho \;R_{r}(t)\right), \end{array}$$

where g(·|m) is the Poisson distribution of mean m, $r_{\mathrm{cap}}\left(t\right)$ the number of rat captured the day t, $f_{\mathrm{cap}}\left(t\right)$ the number of fleas captured the day t, $r_{\mathrm{sero}}\left(t\right)$ the number of seropositive rats among rats captured on day t, ρ the detection rate, and $n_{\mathrm{cap}}$ the number of capture days.

We sampled the posterior distribution of parameters using a Metropolis Markov Chain Monte Carlo (MCMC) algorithm with 400,000 iterations and a burn-in period of 70,000 iterations, employing a log-normal proposal distribution. The SD of the proposal for each parameter was adaptively tuned over 50,000 iterations to achieve an acceptance rate around 0.24. Convergence of the MCMC chains was assessed visually. A summary of fixed and fitted parameters is given in *SI Appendix*, Table S3.

### Evaluation of the Models.

The predictive performance of models was evaluated by computing approximations of the leave-one-out cross-validation (LOO-CV) using Pareto smoothed importance sampling with the *loo* R package (28). LOO-CV assesses a model’s predictive performance by iteratively excluding a single observation, either a daily count of captured rats, captured fleas, or seropositive rats, or a monthly count of human cases fitting the model on the remaining data, and evaluating its prediction accuracy on the omitted data point. Lower LOO-CV scores indicate superior predictive performance (*SI Appendix*, Table S1).

### Interventions.

We investigated three intervention strategies to mitigate the spread of bubonic plague in humans, where rat or flea populations are reduced by a factor c=0.2,0.5,0.8:

1. Reducing the rat population without targeting fleas: This scenario reflects cases where control methods targeting only rats, such as the use of rodenticide or traps like snap traps, are implemented, leaving their fleas free to seek out new hosts. In the model, at the time of intervention $t_{i}$, the rat population $N_{r}\left(t_{i}\right)$ is reduced to ${(1-c)N}_{r}\left(t_{i}\right)$, where c represents the reduction factor.
2. Flea control directly: This approach involves using methods such as insecticides to specifically target flea populations. In the model, the hosted flea population $H(t_{i})$ and free infected flea population $F(t_{i})$ are both reduced to $\left(1-c\right)H\left(t_{i}\right)$ and $\left(1-c\right)F\left(t_{i}\right)$.
3. Simultaneously reducing the rat population and the fleas they host: This can be achieved, for example, by live-capturing rats using live traps, followed by the simultaneous collection of their fleas, thereby preventing their escape, or using a combination of insecticide and rodenticide. In the model, the hosted flea population $H(t_{i})$ and the rat population $N_{r}\left(t_{i}\right)$ are both reduced to $\left(1-c\right)H\left(t_{i}\right)$ and $\left(1-c\right)N_{r}\left(t_{i}\right)$.

For each intervention, we ran 500 simulations and calculated the mean reduction in human plague cases along with the corresponding credible intervals.

### Field Sampling and Ethics Statements.

The capture and handling of terrestrial small mammals were carried out by certified staff of the Plague Unit (Institut Pasteur de Madagascar) in accordance with the national laws, the guidelines of the American Society of Mammalogists (29), and the Directive 2010/63/EU of the European Parliament. To describe the dynamics of human plague cases in Madagascar, we used aggregated routine plague surveillance records collected as part of the national surveillance program in which the reporting of suspected human cases by health centers is mandatory. The secondary use of such surveillance records does not require ethical approval.

## Supplementary Material

Appendix 01 (PDF)

## Data Availability

Capture data, surveillance data, and the code used to produce the results have been deposited in https://gitlab.pasteur.fr/mmmi-pasteur/plague (30).
