# Supplementary material for: Unraveling the role of rat and flea population dynamics on the seasonality of plague epidemics in Madagascar
Source: Proc Natl Acad Sci U S A. 2025 Jun 12;122(24):e2502161122. doi: 10.1073/pnas.2502161122 (PMC12184415; doi:10.1073/pnas.2502161122)
Supplement: Supplementary file 1 — Appendix 01 (PDF) [file pnas.2502161122.sapp.pdf]

Supporting Information for

Unraveling the role of rat and flea population dynamics on the seasonality of plague epidemics in Madagascar

Fanohinjanaharinirina RASOAMALALA, Beza RAMASINDRAZANA, Mamionah J. PARANY, Soloandry RAHAJANDRAIBE, Lovasoa RANDRIANTSEHENO, Soanandrasana RAHELINIRINA, Olivier GORGÉ, Eric VALADE, Mireille HARIMALALA, Minoarisoa RAJERISON*, Simon CAUCHEMEZ[$,*], Antoine BRAULT[$]

$These authors are joint senior authors on this work

*Corresponding authors:

Minoarisoa Rajerison (mino@pasteur.mg), PO. Box 1274 Ambatofotsikely, Antananarivo 101, Madagascar, Phone: +261209741272.

Simon Cauchemez (simon.cauchemez@pasteur.fr), 28 rue du Docteur Roux, 75015 Paris, France, Phone: +33144389253.

This PDF file includes:

Supporting text

Tables S1 to S3

Figures S1 to S2

Model	Leave-one-out cross-validation
No seasonality (Model 1)	4951
Seasonality in rat population (Model 2)	4465
Seasonality in flea population (Model 3)	4209
Seasonality in rat and flea populations (Model 4)	4028
Mass action model with seasonality in rat and flea populations (Model 5)	4025

Table S1. Model comparison. The table compares various models used in the analysis. The Model column specifies the model type as follows. The Leave-One-Out Cross-Validation column presents the values computed using Pareto smoothed importance sampling, where lower values indicate a better model.

Intervention start	Population reduction	Targeting rat population (95% CI)	Targeting flea population (95% CI)	Targeting rat and hosted flea populations (95% CI)
July	20%	8% (-15%, 32%)	14% (-8%, 32%)	22% (2%, 43%)
August	20%	7% (-20%, 30%)	14% (-16%, 35%)	22% (-2%, 43%)
September	20%	8% (-16%, 30%)	14% (-10%, 33%)	22% (0%, 42%)
October	20%	7% (-16%, 31%)	13% (-9%, 35%)	20% (-1%, 39%)
November	20%	7% (-17%, 30%)	12% (-11%, 33%)	19% (-5%, 39%)
December	20%	4% (-21%, 28%)	10% (-16%, 33%)	15% (-11%, 35%)
January	20%	1% (-30%, 26%)	6% (-18%, 33%)	8% (-20%, 30%)
February	20%	1% (-26%, 23%)	3% (-24%, 25%)	5% (-21%, 28%)
March	20%	-1% (-28%, 21%)	1% (-23%, 26%)	1% (-22%, 26%)
July	50%	23% (1%, 41%)	38% (22%, 55%)	53% (39%, 66%)
August	50%	23% (3%, 44%)	38% (17%, 55%)	54% (41%, 69%)
September	50%	23% (3%, 43%)	37% (21%, 56%)	53% (38%, 67%)
October	50%	22% (1%, 41%)	36% (18%, 55%)	51% (36%, 64%)
November	50%	19% (-5%, 39%)	33% (15%, 51%)	46% (30%, 61%)
December	50%	13% (-12%, 34%)	27% (6%, 45%)	37% (14%, 54%)
January	50%	7% (-17%, 32%)	18% (-5%, 38%)	24% (3%, 43%)
February	50%	3% (-23%, 28%)	10% (-13%, 32%)	13% (-10%, 33%)
March	50%	1% (-24%, 24%)	3% (-22%, 29%)	5% (-23%, 25%)
July	80%	45% (28%, 61%)	64% (51%, 76%)	83% (75%, 91%)
August	80%	45% (30%, 61%)	64% (51%, 75%)	83% (75%, 91%)
September	80%	44% (28%, 60%)	63% (51%, 77%)	81% (72%, 89%)
October	80%	41% (25%, 61%)	61% (49%, 74%)	78% (68%, 86%)
November	80%	35% (18%, 55%)	56% (43%, 70%)	72% (61%, 82%)
December	80%	25% (3%, 44%)	46% (30%, 61%)	57% (42%, 70%)
January	80%	14% (-8%, 37%)	31% (11%, 49%)	38% (17%, 55%)
February	80%	6% (-17%, 29%)	17% (-3%, 36%)	20% (-1%, 39%)
March	80%	2% (-22%, 29%)	7% (-20%, 31%)	9% (-14%, 31%)

Table S2. Impact flea and rat control on human plague cases. Reduction in human plague cases (third, fourth, and fifth columns represent different intervention types) is shown with respect to the month of intervention (first column) and the reduction in the targeted population (second column). The 95% credible intervals are provided in parentheses.

Parameter	Value (95% CI)	Definition	References
t_r	172 (161, 182) days	Rodent capacity peak	Fitted
A_r	1.16 (0.93, 1.4)	Relative amplitude of rat capacity	Fitted
B_r	15000	Minimum of rat capacity	Fixed
$1/\phi$	10 days	Average lifespan of an infected flea	Fixed (1, 2)
β	8.02e-6 (7.27e-6, 8.78e-6)	Transmission rate of fleas to rats	Fitted
η	0.5	Infection fatality ratio of rodent	Fixed (3)
$1/\omega$	365.25 days	Average duration of immunity in rats	Fixed (4)
ρ	0.001 (0.0009, 0.0011)	Detection rate	Fitted
t_f	269 (265, 273) days	Flea capacity peak	Fitted
A_f	1.44 (1.24, 1.65)	Relative amplitude of flea capacity	Fitted
B_f	14900 (13400, 16700)	Minimum of flea capacity	Fitted
$1/\gamma$	7 days	Average duration of infectiousness in rats	Fixed (4)
r_r	34 year^{-1}	Rodent population growth rate	Fixed (4, 5)
r_f	138 year^{-1}	Flea population growth rate	Fixed (6)

a	0.000460 (1.67e-4, 0.00110)	Transmission rate fleas to human	Fitted

Table S3. Parameters of the mass-action model. The values indicated are either fitted by the model or fixed according to the literature.

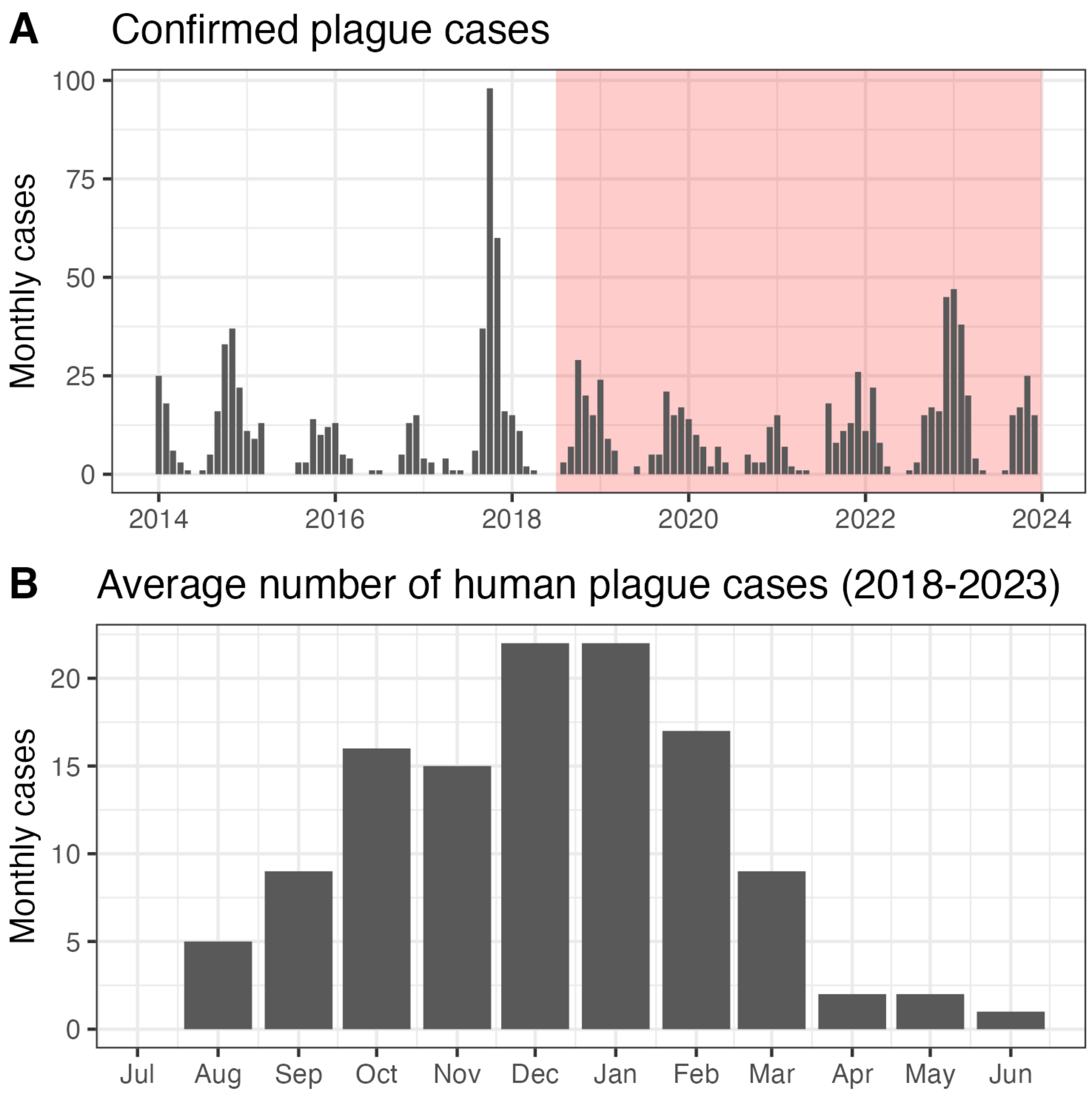

Figure S1. Human plague cases in Madagascar. (A) Number of human plague cases reported each year in plague outbreaks in Madagascar from 2014 to 2023. The red rectangle highlights the period used to compute the monthly average of cases shown in figure B. **(B)** Monthly average number of human plague cases in Madagascar between July 2018 and December 2023.

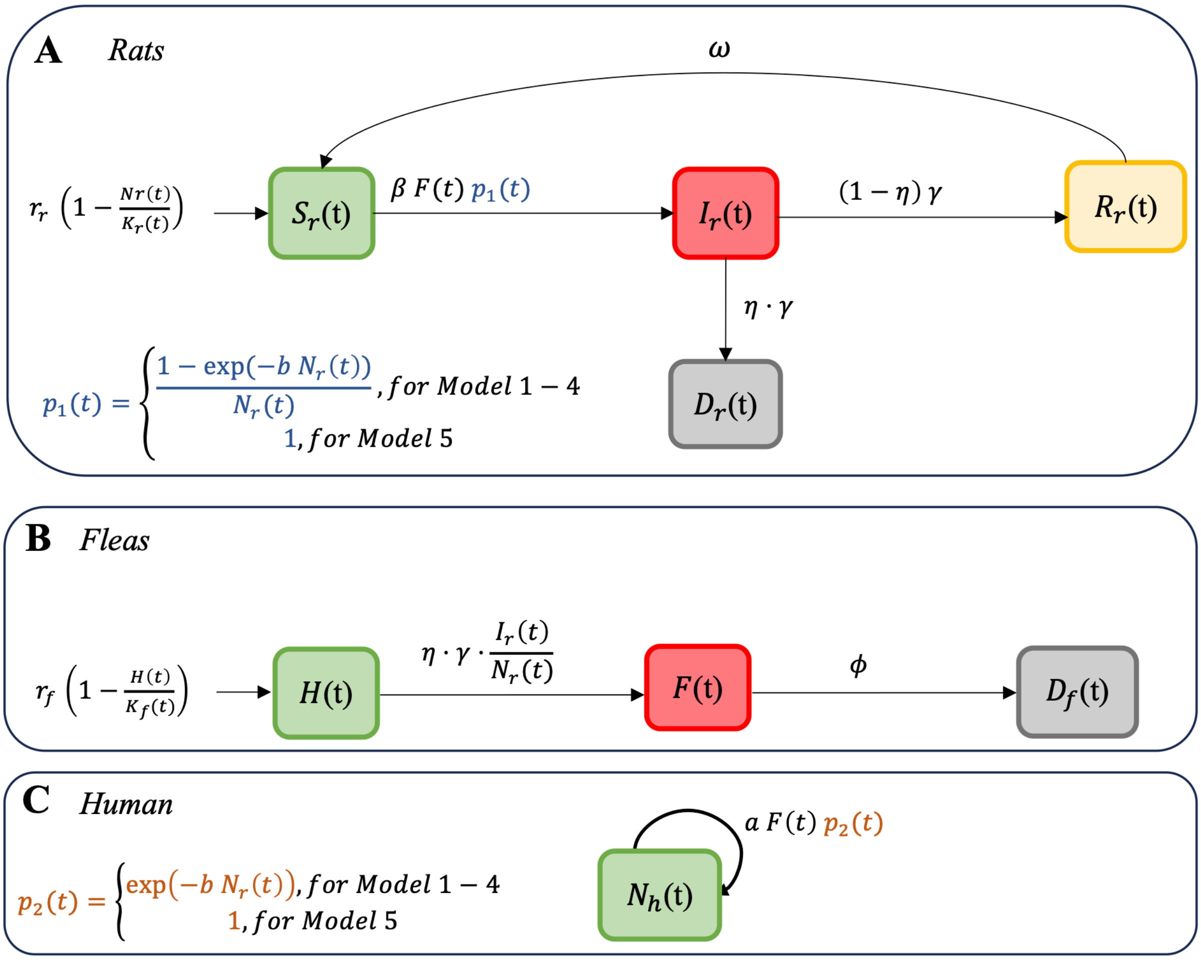

Figure S2. Diagram illustrating a compartmental model of plague transmission among rats, fleas, and humans. (A) The rat population is categorized into susceptible (S_r), infected (I_r), recovered (R_r), and dead following plague infection (D_r). The total number of rats is denoted by N_r. **(B)** The flea population is divided into two groups: fleas on host rats (H) and infected free fleas (F), which are released following the death of an infected rat, and dead fleas (D_f) **(C)** The human compartment is represented by N_h, where humans are infected when exposed to infected free fleas.

SI References

1. A. W. Bacot, LXXX. Observations on the length of time that fleas (Ceratophyllus fasciatus) carrying Bacillus pestis in their alimentary canals are able to survive in the absence of a host and retain the power to re-infect with plague. *J. Hyg. (Lond.)* **14**, 770–773 (1915).

2. M. J. Keeling, C. A. Gilligan, Metapopulation dynamics of bubonic plague. *Nature* **407**, 903–906 (2000).

3. C. Tollenaere, *et al.*, Susceptibility to Yersinia pestis experimental infection in wild Rattus rattus, reservoir of plague in Madagascar. *Ecohealth* **7**, 242–247 (2010).

4. V. Andrianaivoarimanana, *et al.*, Immune responses to plague infection in wild Rattus rattus, in Madagascar: a role in foci persistence? *PLoS One* **7**, e38630 (2012).

5. S. Sridhara, T. R. Krishnamurthy, Population dynamics of Rattus rattus in poultry and implications for control. *Proceedings of the Vertebrate Pest Conference* **15** (1992).

6. D. M. Bland, L. D. Brown, C. O. Jarret, B. J. Hinnebusch, K. R. Macaluso, Methods in Flea Research. *National Institutes of Health* 1–57 (2011).
